# Association between Neuron-Specific Enolase Gene Polymorphism and Delayed Encephalopathy after Acute Carbon Monoxide Poisoning

**DOI:** 10.1155/2020/8819210

**Published:** 2020-10-14

**Authors:** Linlin Xu, Xuejiao Liu, Jing Zhao, Jiao Zeng, Jiapeng Gu, Xiaoli Zhang, Fan Zhang, Yongkai Han, Wenqiang Li, Ping Zhang, Renjun Gu

**Affiliations:** ^1^The Second Affiliated Hospital of Xinxiang Medical University, Henan Mental Hospital, 453002, China; ^2^Henan Key Lab of Biological Psychiatry of Xinxiang Medical University, International Joint Research Laboratory for Psychiatry and Neuroscience of Henan, 453002, China

## Abstract

**Objective:**

The aim of this study is to explore the relationship between neuron-specific enolase (NSE) gene polymorphism and delayed encephalopathy after acute carbon monoxide poisoning (DEACMP) and provide a theoretical basis for DEACMP pathogenesis, diagnosis, and prognosis.

**Methods:**

To investigate this relationship, we screened 6 NSE single nucleotide polymorphisms (SNPs), based on the results of the previous genome-wide association studies (GWAS). A total of 1,201 patients, including 416 in the DEACMP group and 785 in the acute carbon monoxide poisoning (ACMP) group, were detected by the Sequenom MassARRAY® method. The genotype frequencies and alleles of the 6 NSE SNPs (rs2071074, rs2071417, rs2071419, rs11064464, rs11064465, and rs3213434) were compared using different genetic models.

**Results:**

In the SNPs rs2071419 and rs3213434, we found that the genotypes and allele frequencies in the two groups significantly correlated with the grouping of patients (*χ*^2^ = 6.596, *p* = 0.037; *χ*^2^ = 8.769, *p* = 0.012). The haplotypes GGTTTC and CCTTTC of ACMP and DEACMP were different (*χ*^2^ = 6.563, *p* = 0.010; *χ*^2^ = 4.151, *p* = 0.042). We also observed that rs2071419 and rs3213434 significantly correlated with DEACMP-increased risk in the dominant, codominant, and overdominant genetic models. In addition, we speculated that the C allele of the rs2071419 polymorphism and the T allele of the rs3213434 polymorphism in NSE may increase the DEACMP risk (*p* = 0.011, *p* = 0.006).

**Conclusions:**

The results show that rs2071419 and rs3213434 are susceptible sites of DEACMP. The NSE C allele of rs2071419 and T allele of rs3213434 and the haplotypes GGTTTC and CCTTTC may be risk factors for DEACMP.

## 1. Introduction

Carbon monoxide (CO) poisoning is the most common health problem in many countries [1]. Delayed encephalopathy after acute carbon monoxide poisoning (DEACMP) is one of its most common complications [2], which has an incidence rate ranging between 0.2% and 47% [3]. After 2-60 days of “false recovery period” from acute carbon monoxide poisoning (ACMP), neuropsychiatric disorders appear, including dementia, convulsion, chronic headache, and pyramidal and extrapyramidal disorders [3, 4]. Epidemiological studies have found that 10%-30% of ACMP patients have relapse of mental or neurological symptoms, including emotional loss of control; memory, behavioral, and cognitive disorders; and even severe dementia, which are common causes of neurological complications [5].

DEACMP occurrence is related to several factors, such as ischemia, hypoxia, cytotoxic injury, ischemia-reperfusion injury, immune dysfunction, and neurotransmitter imbalance, which lead to the DEACMP overlapping process [3, 6]. DEACMP is common in the clinical practice, and its pathological changes are associated with an extensive demyelination and partial neuron loss in brain white matter and an axonal degeneration and necrosis which mainly involve regions such as the white matter, globus pallidus, thalamus, striatum, and basal ganglia [7]. The exact pathogenesis of the disease is unclear, and it is generally believed to be the result of a combination of multiple factors, including carbon monoxide-induced direct and indirect damages, the theory of tissue hypoxia ischemia-reperfusion injury and microthrombosis [8], the mechanism of nitric oxide injury [9], the abnormal metabolism of neurotransmitters and abnormal signal transduction pathways [10], neuroinflammation, immune injury [11], and individual mechanics of inheritance [12]. In previous studies, it was found that the protein expression levels of neuron-specific enolase (NSE), myelin basic protein (MBP), TNF-*α*, interleukin, and other immune cytokines in serum and cerebrospinal fluid were significantly abnormal in DEACMP development [13–16]. However, there was a significant difference in the probability of DEACMP in patients with similar age, gender, and poisoning degree, such as couples, suggesting that there are differences among different individuals, which may involve individual genetic factors. To explore the relationship between delayed encephalopathy (DEACMP) and neuron-specific enolase (NSE) after ACMP, NSE levels in serum and cerebrospinal fluid (CSF) were measured in an elderly DEACMP group, nonelderly DEACMP group, and control group. The results showed that NSE levels in the serum and cerebrospinal fluid of patients in the DEACMP group were significantly increased (*p* < 0.05) and that the elderly patients in the DEACMP group had significantly higher NSE levels compared to those in the nonelderly DEACMP group. Meanwhile, it was also found that NSE levels in the serum and cerebrospinal fluid of the elderly DEACMP group were significantly lower before discharge compared to its levels at the time of admission and after conventional treatment with neuronal cell activator, vasodilator agent, hyperbaric oxygen chamber, and/or ultraviolet radiation oxygenation. However, these levels in the elderly DEACMP group significantly and negatively correlated at admission [14]. Nonetheless, no analysis of the association between NSE and DEACMP has been reported.

In recent years, Sequenom MassARRAY® has been proved to be an effective method for screening genetic susceptibility genes through SNP genotyping [17]. In the early stage of this study, an Illumina 660W Quad genome-wide SNP typing chip was used to complete the genome-wide SNP gene-wide association studies (GWAS) of peripheral blood samples of 175 DEACMP and 244 ACMP patients in China [18]. Allele frequencies of all SNPs were compared between delayed encephalopathy after ACMP and control groups and then ranked. A total of 123 SNPs gave an OR > 1.4, and of these, 46 were mapped in or close to known genes. Forty-eight SNPs located in 19 genes were associated with DEACMP after correction for 5% FDR in the genome-wide association of pooled DNA. In subsequent studies, we used techniques such as GWAS and PCR-RFLP to verify the susceptibility of several DEACMP-related genes, such as NRXN3. Studies have found that genes, such as NRXN3, may be related to DEACMP and that obvious abnormalities in nerve injury factors, such as NSE, have also been found in the serum and cerebrospinal fluid of DEACMP patients. Although the mechanism is not clear, the study of DEACMP has become a hot research topic from the perspective of molecular genetics. Then, we set out to study the association between NSE gene polymorphism and DEACMP. Genomic DNA was extracted from 416 patients in the DEACMP group and from 785 patients in the ACMP group, and Sequenom detection was performed. In this study, we investigated whether several NSE SNPs affect the occurrence of DEACMP. For this, an Illumina 660W Quad genome-wide SNP typing chip was used for screening and 28 sites with high risk were selected based on the above results, and 26 sites were selected by evaluation for the Sequenom detection. Finally, 26 SNP26 SNPs loci were successfully detected in all patients in the DEACMP and ACMP groups, including LR1B, PD4, CHR2, NSE, and LRCH genes. Therefore, in this study, the Sequenom method was used to further verify 6 candidate NSE gene polymorphisms in DEACMP and ACMP patients. This study explored the relationship between NSE gene polymorphism and DEACMP genetic susceptibility and provided theoretical basis for DEACMP pathogenesis, diagnosis, and prognosis.

## 2. Materials and Methods

### 2.1. Subjects

The Chinese diagnostic standard of management of occupational ACMP (GBZ23-2002) in DEACMP clinical diagnosis standard is as follows: after recovery of ACMP patients from the consciousness disturbance, which lasted approximately 2–60 days (latency phase), one of the following clinical manifestations was observed: (1) spirit and consciousness obstacles: a state of dementia and delirium; (2)extrapyramidal neurological disorders: Parkinson's syndrome; (3) nerve injury of cone system, such as hemiplegia and positive pathological reflex; and (4) cerebral cortex focal sexual dysfunction, such as aphasia, blindness, or secondary epilepsy. Brain CT or MRI examination found symmetric lesions of the bilateral globus pallidus and extensive demyelination changes of the cerebral white matter. Electroencephalogram (EEG) examination may detect moderate and highly generalized abnormalities. From November 2006 to April 2019, a total of 1,201 patients (416 patients in the DEACMP group and 785 patients in the ACMP group) were included from the emergency and neurology departments of 11 hospitals, including the Second Affiliated Hospital of Xinxiang Medical University and the First Affiliated Hospital of Xinxiang Medical University. In view of the extremely rare DEACMP cases in the under 30-year-old population and to reach a relative match, the age limits of the study subjects were all set as over 40 years old. Patients with the following conditions were excluded: (1) received immunosuppressive and hormone therapies; (2) received vaccination within the past 6 months; (3) history of allergy; (4) diagnosed with a central nervous system disease, serious heart illness, liver and kidneys diseases or dysfunctions, serious diabetes, endocrine disease, immune system disease, malignant tumors, malnutrition, alcohol dependence, or other mental disorders; (5) infection occurred within the past 2 weeks; and (6) pregnancy, lactation, and menstruation. DEACMP false healing period can last up to 60 days; therefore, all ACMP patients were followed up for more than 90 days. The subjects were divided into two groups based on DEACMP occurrence. If DEACMP occurred, the subject was assigned to the DEACMP group, and if not, the subject was assigned to the ACMP group. The research plan was approved by the ethics committee of the Second Affiliated Hospital of Xinxiang Medical University and the ethics committee of all participating hospitals and research institutes, and all participants provided written informed consents prior to any study-related procedures. The average age, gender, and education level of patients in the DEACMP and ACMP groups matched. The peripheral blood samples of DEACMP patients were collected into anticoagulant vacuum tubes between 6:00 and 8:00 a.m. after overnight fasting. Using the same method, blood samples of the ACMP patients were collected within 24 hours after the subjects were fully awake. All blood samples were labelled and stored at -80°C.

### 2.2. Individual Genotyping

Genomic DNA was extracted from the peripheral blood of each patient with the TIANGEN Kit (DP304, Beijing TIANGEN Biotechnology Co., Ltd.). The SNP genotyping method for all samples was based on the Sequenom MassARRAY® platform, and all amplification primers of the 6 NSE gene SNPs were designed according to the dbSNP database for sequencing and amplified by multiplex PCR to obtain site-specific PCR. The initial multiplex PCR amplification was performed using ABI Veriti-384 PCR. SAP (shrimp alkaline phosphatase) was used to remove the free dNTPs from the reaction system. After SAP treatment, the extended mixture was added in the abovementioned PCR system, to carry out single-base extension reactions, and then, a resin purification was conducted. Finally, a MassARRAY Nanodispenser (Agena, Inc.) was used to transfer the PCR products to the 384-well SpectroCHIP® bioarray. The genotypes and alleles were detected by the mass spectrometer MassARRAY Analyzer 4.0 and analyzed by the mass spectrometer MassARRAY TYPER4.0. According to the results of the analyses, the 6 SNPs were separately genotyped in all the samples.

### 2.3. Statistical Analysis

The SPSS 20.0 software was used for data analyses. An independent-sample *t*-test was used for age comparison between the two groups, a crosstable was used for describing the patients' educational level, and gender and chi-squared analyses were used for comparisons between the two groups. The distribution of genotypes, according to Hardy-Weinberg equilibrium law, was tested by goodness-of-fit chi-square, and the correlation analysis was conducted by a binary logistic regression analysis test. The allele frequency and genotype distribution were also tested by binary logistic regression analyses. Binary logistic regression analyses were used in genetic model analyses, and the overall statistical significance level was set at *p* < 0.05.

## 3. Results

### 3.1. Clinical Characteristics

The physical location and gene frequency of the 6 NSE gene SNPs (rs2071074, rs2071417, rs2071419, rs11064464, rs11064465, and rs3213434) are shown in Table 1. The number, age, gender, and education level of the two groups are shown in Table 2. For the 6 NSE gene SNPs, rs2071074 (C/G), rs2071417 (C/G), rs2071419 (C/T), rs11064464 (C/T), rs11064465 (A/T), and rs3213434 (C/T), different numbers of test results were separately obtained: 1,163 (DEACMP: 416; ACMP: 747), 1,158 (DEACMP: 413; ACMP: 745), 1,152 (DEACMP: 415; ACMP: 737), 1,158 (DEACMP: 414; ACMP:744), 1,163 (DEACMP: 416; ACMP: 747), and 1,165 (DEACMP: 416; ACMP: 749). In addition, the demographic data matched between the two groups in rs2071074 (average age: *p* = 0.227; gender distribution: *p* = 0.176; education level: *p* = 0.085), rs2071417 (average age: *p* = 0.189; gender distribution: *p* = 0.295; education level: *p* = 0.095), rs2071419 (average age: *p* = 0.134; gender distribution: *p* = 0.294; education level: *p* = 0.063), rs11064464 (average age: *p* = 0.184; gender distribution: *p* = 0.351; education level: *p* = 0.100), rs11064465 (average age: *p* = 0.236; gender distribution: *p* = 0.291; education level: *p* = 0.091), and rs3213434 (average age: *p* = 0.262; gender distribution: *p* = 0.284; education level: *p* = 0.087). It is worth noting that the genotype distribution of these 6 SNPs, between the two groups, conformed to the Hardy-Weinberg equilibrium law (in all cases, *p* > 0.05, Table 3).

### 3.2. Analyses of the Association between the 6 SNP Polymorphisms and DEACMP

**Figure 1:**
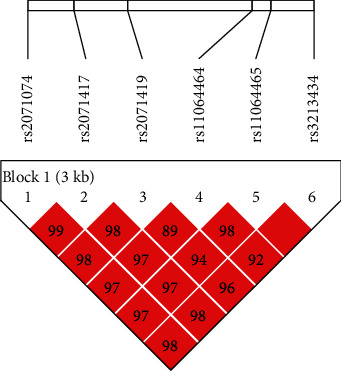
Haplotype block structure of ACMP and DEACMP patients. The index association SNP is represented as a diamond. The color of the remaining SNPs (circles) represents LD, and the indexed SNPs are based on the paired *r*^2^ values in our data.

The analyses of the association between rs2071074, rs2071417, rs2071419, rs11064464, rs11064465, and rs32134346 SNP polymorphisms and DEACMP and its increased risk under different genetic models are shown in Table 4. The allele frequencies of rs2071074, rs2071417, rs11064464, and rs11064465 in the two groups were similar, but there was no significant difference (*p* > 0.05). Rs2071074, rs2071417, rs2071419, rs11064464, rs11064465, and rs3213434 polymorphisms in the recessive model and rs2071419 and rs3213434 polymorphisms in the codominant model were insignificantly related to DEACMP-increased risk (*p* > 0.05). The analysis of the correlation between rs2071419 and DEACMP showed that rs2071419 is related to DEACMP susceptibility in the codominant model (TT vs. TC, OR = 0.732, 95% CI: 0.562-0.953, *p* = 0.020), the dominant model (TT vs. TC+CC, OR = 0.722, 95% CI: 0.561-0.928, *p* = 0.011), and the overdominant model (TT+CC vs. TC, OR = 0.755, 95% CI: 0.582-0.980, *p* = 0.035). Similarly, rs3213434 was associated with DEACMP sensitivity in the codominant model (CC vs. CT, OR = 0.681, 95% CI: 0.523–0.887, *p* = 0.004), the dominant model (CC vs CT+TT, OR = 0.684, 95% CI: 0.532–0.880, *p* = 0.003), and the overdominant model (CC+TT vs. TC, OR = 0.698, 95% CI: 0.538-0.906, *p* = 0.007). In DEACMP patients, the differences in the relative distribution of the rs2071419 allele frequency were statistically significant (*p* = 0.011, OR = 0.760, 95% CI: 0.615-0.940). Allele C may be the potential DEACMP risk allele. The relative difference in the distribution of rs3213434 allele frequency was significant (*p* = 0.006, OR = 0.741, 95% CI: 0.599-0.917), and T may be the potential DEACMP risk in this allele. To further analyze the haplotype structures of ACMP and DEACMP, we evaluated the pairwise linkage disequilibrium (LD) of 6 SNPs in the 2 groups using standardized *D*′ and *r*^2^ values and identified haplotypes on the 6 SNPs from both groups. The location, LD structure, and *D*′ values of these SNPs are shown in Figure 1. Six SNPs formed a LD block and produced four haplotypes. The differences of GGTTTC in ACMP and CCTTTC in DEACMP are shown in Table 5 (*χ*^2^ = 6.563, *p* = 0.010; *χ*^2^ = 4.151, *p* = 0.042).

## 4. Discussion

We analyzed 6 NSE polymorphisms (rs2071074, rs2071417, rs2071419, rs11064464, rs11064465, and rs3213434) in the two groups and analyzed their association with DEACMP in different genetic models. The results showed that the NSE polymorphisms, rs2071419 and rs3213434, were related to DEACMP-increased risk in the codominant (TT vs. TC, CC vs. CT), dominant, and overdominant genetic models. In addition, the C allele of the rs2071419 polymorphism, the T allele of the rs3213434 polymorphism, and the haplotypes GGTTTC and CCTTTC may increase DEACMP risk.

Previous studies have shown that the pathogenesis of DEACMP is complex and hard to reveal, but the inflammatory response mechanism that is induced by immune injury can lead to a DEACMP-related neural damage [19, 20]. Pathological studies and CT and MRI examination of the head showed that the main pathological changes of the disease were associated with an extensive demyelination of the brain white matter, a symmetrical softening of the bilateral globus pallidus, and a partial neurons' loss [21]. Our previous studies showed that MBP, NSE, 5-HT, and DA protein levels were significantly abnormal in serum and CSF, suggesting that these biochemical markers existed in the pathogenesis of DEACMP that was mediated brain injury [22, 23].

In addition, increasing evidence has shown that DEACMP has a genetic susceptibility that is due to a combination of the environment and genetics. Wang et al. [24] investigated 405 cases of DEACMP in northern Henan Province and found that for a couple who were CO (carbon monoxide) poisoned during the same period, the one with the mild CO poisoning had DEACMP, while the other did not. For patients with the same extent of CO poisoning, the DEACMP incidence rate was also different. Yamagata and Ishii [12] found that DEACMP may also occur in patients with mild CO poisoning symptoms. It was suggested that individual differences that are determined by genetic factors may be involved in the occurrence of the disease and that the related genes and their polymorphic differences may play important roles. Our group used the Illumina 660W Quad whole-genome SNP genotyping chip to take the lead in completing the analysis of the whole genome SNP gene association (GWAS) of 175 DEACMP patients and 244 ACMP patients' peripheral blood samples in China and the group also screened the SNP sites that were related to DEACMP and identified 441 potential susceptible gene sites with a difference that was greater than 0.5 [18]. On this basis, some positive sites were, respectively, verified. The study confirmed that two SNPs (rs11845632, rs2196447) in axon protein 3 gene (NRXN3) correlated with DEACMP [18] and that there was a correlation between rs1784594 in the Parkin gene and DEACMP. We also found that allele A of rs1784594 may increase the risk of DEACMP in female ACMP patients [25], that LRP1B gene rs1541976 polymorphism is a potential susceptible factor of DEACMP [26], and that LRCH1 (rs1539177, rs17068697, rs9534475, and rs2236592) is associated with DEACMP [27]. These results suggest that some ACMP patients are specifically sensitive to acute carbon monoxide poisoning due to polygenic defects, which leads to the initiation of a central nervous system's autoimmune response that attacks the corresponding target cells, leading to the relevant clinical changes. The results also provide a new theoretical basis for the study of DEACMP pathogenesis, disease diagnosis, treatment, and prevention and lay a solid foundation for optimizing individual diagnosis and treatment.

In this study, six SNP loci of the NSE gene were selected from the candidate loci that were obtained by GWAS in the early stage and that were detected by the Sequenom MassARRAY® genotyping technology. This approach explored the relationship between SNP polymorphism and DEACMP genetic susceptibility and provided clear evidence for the necessity of performing genetic studies on DEACMP pathogenesis. The SNP genotyping results showed that only two of the six NSE SNP polymorphisms (rs2071419, rs3213434) were closely related to DEACMP. NSE, located on chromosome 12p13.31, is an important enzyme that is involved in glycolysis. It has a molecular weight of approximately 80 kD and a variety of dimers, which are composed of 3 subunits *α*, *β*, and *γ*. The *α* subunit is found in many types of mammalian tissues, while the *β* subunit is mainly found in the heart and muscle tissues. The isomers of *αγ* and *γγ* enzymes are called neuron-specific enolase (NSE) or *γ* enzyme, of which *γγ* specifically exists in brain neurons and neuroendocrine cells and accounts for 1.5% of all soluble proteins in the brain. When neurons are damaged or necrotic, NSE overflows into the cerebrospinal fluid from the cells. Since NSE is not contained in glial cells and other nervous tissues, NSE can be used as an objective indicator for assessing neuronal necrosis. NSE is a glycolytic enzyme among different isoenzymes of the enolase family, and its subtype exists in the central nervous system neuronal cytoplasm. When the integrity of the neuronal cell membrane is lost, NSE is thought to be released from neurons and glial tissues into the blood. Since NSE is not physiologically secreted but overflows from neurons when axons are damaged, the serum NSE level can be used as a marker of nerve cell damage in patients with a variety of diseases, including traumatic and hypoxic brain damage, epileptic status, and cardiac arrest [28–32].

It has been reported that serum NSE (measured within 24 hours after ACMP) in the DEACMP group was significantly increased and was an early DEACMP predictor [33]. There are two main mechanisms of NSE release that are caused by neuron cell injury in ACMP: (1) the high affinity of CO to hemoglobin and cell hypoxia [34] and (2) CO exposure that can cause inflammation through independent ischemia-reperfusion injury, synergistic effect of vascular endothelial, oxygen free radical-mediated lipid peroxidation, and nitric oxide (NO) that is released by platelets following carbon monoxide exposure. It has been reported that in the DEACMP group, the number of damaged nerve cells was more than that in the non-DEACMP group and that NSE in the DEACMP group was higher than that in the non-DEACMP group [35–39]. It is worth noting that DEACMP is the most serious complication following ACMP and involves inflammatory factors and several cytokines. Therefore, these findings further support our conjecture that ACMP causes neuron damage and that serum NSE can be used as a DEACMP predictor following ACMP. In this study, we discovered a new association between 6 NSE gene polymorphisms and DEACMP. Our results showed that two NSE polymorphisms (rs2071419 and rs3213434) were associated with an increased DEACMP risk in the codominant (TT vs. TC, CC vs. CT), dominant, and overdominant genetic models. In addition, the C allele of rs2071419 polymorphism and the T allele of rs3213434 polymorphism may increase the DEACMP risk. Overall, the current research shows that DEACMP has a genetic susceptibility that is the result of the interaction between environmental factors and genetic backgrounds. More valuable findings can be expected by expanding the sample size and conducting further research.

## 5. Conclusions

Our results revealed a significant correlation between two NSE polymorphisms (rs2071419, rs3213434) and DEACMP patients. They also demonstrate that the allele C of rs2071419 polymorphism, the allele T of rs3213434 polymorphism, and the haplotypes GGTTTC and CCTTTC may be risk factors for ACMP development into DEACMP. However, the research results are limited to a small part of the Han population in Northern Henan Province and should be confirmed in other populations. Meanwhile, these studies may help in revealing the mechanism of DEACMP following ACMP and the role of NSE in neurodegenerative diseases and provide theoretical basis for future diagnostic and prognostic research.

## Figures and Tables

**Table 1:** Physical locations of the 6 NSE gene SNPs.

Variant	Chrom	Position	MAF
(rs2071074)	12	6916599	0.125(C)
(rs2071417)	12	6917143	0.125(C)
(rs2071419)	12	6917768	0.346(C)
(rs11064464)	12	6919230	0.105(C)
(rs11064465)	12	6919445	0.105(A)
(rs3213434)	12	6919954	0.309(T)

**Table 2:** Demographic variables of DEACMP and ACMP patients genotyped for the 6 SNPs (rs2071074, rs2071417, rs2071419, rs11064464, rs11064465, and rs3213434 polymorphisms).

Characteristics	Number of samples	Age	Sex		Education level		
			Male	Female	Uneducated	Primary school	Middle school
rs2071074							
DEACMP	*N* = 416	64.80 ± 11.69	244	172	130	142	144
ACMP	*N* = 747	63.91 ± 12.19	413	344	192	257	298
Statistics		*t* = 1.209	*χ* ^2^ = 1.828		*χ* ^2^ = 4.933		
*p* value		0.227	0.176		0.085		
rs2071417							
DEACMP	*N* = 413	64.84 ± 11.73	241	172	129	141	143
ACMP	*N* = 745	63.87 ± 12.17	411	334	192	257	296
Statistics		*t* = 1.313	*χ* ^2^ = 1.096		*χ* ^2^ = 4.698		
*p* value		0.189	0.295		0.095		
rs2071419							
DEACMP	*N* = 415	64.81 ± 11.70	243	172	130	142	143
ACMP	*N* = 737	63.71 ± 12.13	408	329	188	253	296
Statistics		*t* = 1.498	*χ* ^2^ = 1.103		*χ* ^2^ = 5.522		
*p* value		0.134	0.294		0.063		
rs11064464							
DEACMP	*N* = 414	64.83 ± 11.72	241	173	130	140	144
ACMP	*N* = 744	63.85 ± 12.17	412	332	193	255	296
Statistics		*t* = 1.328	*χ* ^2^ = 0.870		*χ* ^2^ = 4.611		
*p* value		0.184	0.351		0.100		
rs11064465							
DEACMP	*N* = 415	64.81 ± 11.70	243	172	130	142	143
ACMP	*N* = 748	63.94 ± 12.20	414	334	193	257	298
Statistics		*t* = 1.186	*χ* ^2^ = 1.117		*χ* ^2^ = 4.784		
*p* value		0.236	0.291		0.091		
rs3213434							
DEACMP	*N* = 416	64.80 ± 11.69	244	172	130	142	144
ACMP	*N* = 749	63.98 ± 12.23	415	334	193	257	299
Statistics		*t* = 1.123	*χ* ^2^ = 1.147		*χ* ^2^ = 4.881		
*p* value		0.262	0.284		0.087		

**Table 3:** Results of Hardy-Weinberg equilibrium test for genotype distributions of NSE.

SNPs	Genotypes	Risk allele	Risk allele frequency ACMP/DEACMP	Actual value	Test value	*p* value
rs2071074	CC CG GG	C	0.250/0.222	49/25 276/135 422/256	*χ* ^2^ = 0.182/1.579	*p* = 0.670/0.209
rs2071417	CC CG GG	C	0.251/0.222	47/24 275/135 423/254	*χ* ^2^ = 0.066/1.131	*p* = 0.797/0.287
rs2071419	CC TC TT	C	0.233/0.188	43/18 258/120 436/277	*χ* ^2^ = 0.346/1.154	*p* = 0.556/0.283
rs11064464	CC CT TT	C	0.193/0.175	29/13 229/119 486/282	*χ* ^2^ = 0.097/0.011	*p* = 0.756/0.918
rs11064465	AA TA TT	A	0.192/0.175	25/13 237/119 486/283	*χ* ^2^ = 0.356/0.013	*p* = 0.551/0.909
rs3213434	CC TC TT	T	0.232/0.183	442/282 267/116 40/18	*χ* ^2^ = 0.002/1.826	*p* = 0.969/0.177

**Table 4:** Correlation analysis of NSE polymorphisms under different genetic models and DEACMP risk.

SNPs	Genetic models		DEACMP	ACMP	Pobs	OR (95% CI)
rs2071074	Allele	G/C	647/185	1120/374	0.130	0.856 (0.700, 1.047)
	Codominant	GG/GC/CC	256/135/25	422/276/49	0.102/0.503	0.806 (0.623, 1.043)/0.841 (0.507, 1.395)
	Dominant	GG/GC+CC	256/160	422/325	0.095	0.812 (0.635, 1.037)
	Recessive	GG+GC/CC	391/25	698/49	0.713	0.911 (0.554, 1.498)
	Overdominant	GG+CC/GC	281/135	471/276	0.124	0.820 (0.636, 1.056)
rs2071417	Allele	G/C	643/183	1103/369	0.117	0.851 (0.695, 1.041)
	Codominant	GG/GC/CC	254/135/24	423/275/47	0.126/0.538	0.818 (0.632, 1.058)/0.850 (0.508, 1.424)
	Dominant	GG/GC+CC	254/159	423/322	0.118	0.822 (0.643, 1.051)
	Recessive	GG+GC/CC	389/24	698/47	0.735	0.916 (0.552, 1.521)
	Overdominant	GG+CC/GC	278/135	470/275	0.150	0.830 (0.644, 1.070)
rs2071419	Allele	T/C	674/156	1130/344	0.011	0.760 (0.615, 0.940)
	Codominant	TT/TC/CC	277/120/18	436/258/43	0.020/0.152	0.732 (0.562, 0.953)/0.659 (0.372, 1.166)
	Dominant	TT/TC+CC	277/138	436/301	0.011	0.722 (0.561, 0.928)
	Recessive	TT+TC/CC	397/18	694/43	0.278	0.732 (0.416, 1.286)
	Overdominant	TT+CC/TC	295/120	479/258	0.035	0.755 (0.582, 0.980)
rs11064464	Allele	T/C	683/145	1201/287	0.293	0.888 (0.712, 1.108)
	Codominant	TT/TC/CC	282/119/13	486/229/29	0.416/0.451	0.896 (0.687, 1.168)/0.773 (0.395, 1.510)
	Dominant	TT/TC+CC	282/132	486/258	0.335	0.882 (0.683, 1.139)
	Recessive	TT+TC/CC	401/13	715/29	0.509	0.799 (0.411, 1.555)
	Overdominant	TT+CC/TC	295/119	515/229	0.469	0.907 (0.697, 1.181)
rs11064465	Allele	T/A	685/145	1209/287	0.309	0.892 (0.715, 1.112)
	Codominant	TT/TA/AA	283/119/13	486/237/25	0.272/0.746	0.862 (0.662, 1.123)/0.893 (0.450, 1.773)
	Dominant	TT/TA+AA	283/132	486/262	0.267	0.865 (0.670, 1.117)
	Recessive	TT+TA/AA	402/13	723/25	0.847	0.935 (0.473, 1.848)
	Overdominant	TT+AA/TA	296/119	511/237	0.286	0.867 (0.667, 1.127)
rs3213434	Allele	C/T	680/152	1151/347	0.006	0.741 (0.599, 0.917)
	Codominant	CC/CT/TT	282/116/18	442/267/40	0.004/0.235	0.681 (0.523, 0.887)/0.705 (0.396, 1.255)
	Dominant	CC/CT+TT	282/134	442/307	0.003	0.684 (0.532, 0.880)
	Recessive	CC+CT/TT	398/18	709/40	0.447	0.802 (0.453, 1.417)
	Overdominant	CC+TT/TC	300/116	482/267	0.007	0.698 (0.538, 0.906)

**Table 5:** Haplotypes of ACMP and DEACMP.

Haplotype	Freq.	Case, control ratio counts	Case, control frequencies	Chi-square	*p* value
GGTTTC	0.567	469.4 : 306.6, 1025.4 : 836.6	0.605, 0551	6.563	0.010
GGCTTT	0.2	147.8 : 628.2, 378.7 : 1483.3	0.191, 0.203	0.565	0.452
CCTCAC	0.161	110.5 : 665.5, 313.3 : 1548.7	0.142, 0.168	2.726	0.099
CCTTTC	0.054	31.1 : 744.9, 111.2 : 1750.8	0.040, 0.060	4.151	0.042

## Data Availability

The experimental data used to support the findings of this study are included within the article.
